# Ninth International Medical Congress

**Published:** 1888-02

**Authors:** 


### Ninth International Medical Congress.—XVIIIth Section, Dental and Oral Surgery.

Washington, D. C., September 5-10.

### CLINICS.

The Franklin School Building, corner of 13th and K streets, was devoted to clinics and the exhibition of appliances; also the exhibits of the dental manufacturing companies.

The different rooms in the basement had been fitted with every thing necessary in prosthetic dentistry, and here clinics were given in continuous gum work by Dr. L. P. Haskell, Chicago; Dr. John Allen, New York; and by Dr. J. Hall Lewis, Washington, D. C., in gold plate work.

Dr. W. W. Evans, Washington, D. C., zylonite and celluloid, and Dr. C. C. Carroll, Meadeville, Pa., in cast aluminum work.

Dr. J. J. R. Patrick, also had a bench for exhibiting his method of making gold crown and bridge work.

From the school rooms on the first floor all the desks and benches had been removed and twenty-four operating chairs, etc., supplied by the S. S. White Company, distributed through the different rooms. Around each chair a low railing had been placed, protecting operator and patient from crowding spectators, though near enough to allow a close inspection of the work. Raised platforms with a series of steps to accommodate rows of observers standing behind and above the others afforded opportunity for large numbers to watch the operators with the greatest facility. These arrangements were all planned and carried out by Dr. R. Finley Hunt, Washington.

On the second floor were found the displays of the S. S. White Co.; the Welch Dental Co.; R. S. Williams, the American Dental Manfg. Co.; the Wilmington Manfg. Co., and others. Seabury & Johnson also had a fine display of their dental specialties, including their various medicated absorbent cottons and lint, fine rubber dam, hydro-napthol in various sizes and styles of packages, also their liquid dentifrice, *Eau Favorite*, and the Handicap Tooth Powder.

The water motor of Dr. Campbell, Dundee, Scotland, could not be exhibited in this building for lack of sufficient water power. It was therefore exhibited in the rear court yard of the Arlington hotel, where it attracted considerable attention.

Among the appliances exhibited were the interdental splints of Dr. Wm. Carr, New York, for the treatment of maxillary fractures; a bandage for the correction of certain irregularities by Dr. V. E. Turner, Raleigh, N. C.; a remarkable collection of casts, models, and appliances for regulating, by Dr. V. H. Jackson, New York; a cleft palate obturator and nasal support, by Dr. W. B. McLeod, Edinburg, Scotland, and Dr. Geneve's anatomical articulator, syphon, and speculum.

Dr. J. J. R. Patrick had a new catch for the rubber dam by which a fresh piece of tape can be slipped in for each patient, in place of the soiled elastic generally in use. He had also a gum-shield for protecting the gums while trimming roots for applying crowns.

Dr. T. S. Waters had German silver caps for slipping over teeth with finished gold fillings or crowns, protecting them from injury while filling adjacent cavities.

Dr. Butler showed Dr. Palmer's rubber dam forceps for punching the dam and slipping it over additional teeth, when already adjusted on others in the mouth.

Among the operators in crown and bridge-work were Drs. H. A. Parr, New York, whose gold crowns have removable porcelain fronts; Dr. E. B. Call, Peoria, Ill., solid gold crowns; Dr. H. C. Merriam, Salem, Mass., who also illustrated his method of dry-grinding for porcelain; Dr. T. S. Waters, Baltimore, Md.; Dr. E. Parmley Brown, Flushing, N. Y.; Dr. J. R. Knapp, New Orleans, La.; Dr. S. S. Stowell, Pittsfield, Mass.; Dr. E. T. Starr, Philadelphia, Pa,; Dr. I. S. Thompson, Atlanta, Ga., using the How crown in bridge-work, and Dr. E. S. Ludwig, Chicago.

Dr. J. A. Daly was present with Daly's all-gold lining for vulcanite plates.

But little was done towards clinics on Monday beyond examining patients and making appointments.

On Tuesday, September 9, Dr. Louis Ottofy, Chicago, implanted *a la Younger*, a superior central incisor, where the tooth had been out seven years.

Dr. Geo. H. McCausey (Janesville, Wis.) treated a tooth with gangrenous pulp, injecting per-oxide of hydrogen, until all putrescent matter was removed, and then treating with a $\frac{1}{1000}$ solution of hydro-napthol in distilled water as a germicide, filling temporarily with phosphate cement. By this treatment the use of pulp canal instruments is avoided, contact of low organisms with the peridental membrane prevented, and subsequent inflammation avoided.

He also treated a tooth of which the vitality had been destroyed by Dr. Matteson (Chicago) with dogwood.

Two of the roots which Dr. McCausey treated were, at a subsequent clinic, crowned by Dr. H. A. Parr.

Dr. J. P. Geran (Brooklyn, N. Y.) with cylinders, soft gold, and the Herbst method, using Bonwill's mechanical mallet and an automatic hand mallet, filled a very large cavity in the left superior second molar, involving the whole of the masticating surface, and building up one-fourth of the tooth, using 66 grains of gold.

He also pointed out in the same mouth (a dentist's) a very large filling in the mesial and grinding surface of the right inferior first molar, filled last November, by the Herbst method, of which the surface is still well polished and the edges perfect, and which has given great satisfaction.

Dr. W. N. Conrad (St. Louis) destroyed a fungus growth in the pulp chamber of a right superior molar, finding live pulp in the canals, which he carbolized and removed, filling immediately.

Dr. T. D. Shumway (Plymouth, Mass.) illustrated the use of his ivory-points. The instrument which he used was a double-ender having at one end a gold point with which he tore off little rags of cohesive foil from a leaf laid on a velvet pad on his knee. Passing it through the flame of the alcohol lamp, he conveys it to the cavity on the gold point, and burnishes it down with the ivory point on the other end of the handle.

Dr. J. W. Wassall (Chicago) filled the root canals of a second lower molar with gutta-percha in solution and cones, using gold wire in the smallest canals.

Dr. G. S. Salomon (Chicago) using the Detroit Motor Co. engine, the Bonwill-Webb mallet, and Kearsing's foil, filled the distal surface of a central incisor, shoeing the cutting edge.

Dr. R. F. Ludwig (Chicago) with Miss McLaughlin as assistant, and using the Whitefield electric engine (Evanston, Ill.) and a very heavy steel mallet, contoured the anterior surface of a second superior bicuspid, using both cohesive and non-cohesive gold.

Dr. Wm. Crenshaw (Atlanta, Ga.) began a series of connective contouring operations.

Dr. H. A. Parr (New York) demonstrated the value of his universal separator, in the mouth of Dr. C. P. Dennis, of Portsmouth, Ohio, a man of fifty years of age with an unbroken row of solidly-planted massive teeth offering the best possible test of the power of a separator. The teeth were without exception, from molars to incisors, separated in less than three minutes each, Dr. Dennis testifying that it was much less painful than the rubber block or wooden wedge, and left no soreness whatever.

Dr. R. B. Adair (Gainesville, Ga.) began the treatment of two cases of Pyorrhea Alveolaris.

CLINICS.

WEDNESDAY, September 7.

Dr. R. H. Hofheintz (Rochester, N. Y.) not having found a patient for his promised contour operation, filled a posterior proximal cavity in a second bicuspid, with cylinders.

Dr. M. C. Marshall (Little Rock, Ark.) could not obtain the "velvet cylinders" he was on the programme for, but used William's gold in making a compound filling in a superior central incisor.

Dr. H. F. Harvey (Cleveland, Ohio) contoured superior central incisors, proximal cavities, using gold and platinum foil.

Dr. W. J. Younger (San Francisco, Cal.) gave a clinic on his well-known method of implantation.

Dr. J. J. R. Patrick crowned a first bicuspid.

Dr. Geo. W. Whitefield (Evanston, Ill.) gave a clinic with "Steurer's Plastic Gold" contouring the mesial surface of the right superior lateral incisor, in fifteen minutes; he also contoured the mesial surface of the second bicuspid, using his electric engine bracket in these operations.

Dr. Morris E. Smith (Chicago) operating upon Dr. Sidney Stowell (Pittsfield, Mass.) filled an anterior proximal cavity in a molar with soft gold.

Dr. G. H. Chewing (Fredricksburg, Va.) made a contour filling with hand pressure, using quarter century foil.

Drs. Crenshaw and Adair continued the operations of Tuesday.

THURSDAY, September 8.

At 8 A. M. Wednesday and Thursday, in the basement, Dr. C. C. Carroll (Meadville, Pa.) exhibited his process of casting aluminum for plates and bridge-work. At 2 P. M. Thursday in the microscopical room he began a clinical lecture on his method. His apparatus consists of an automatic gas or gasoline furnace, pneumatic plumbago crucibles with special iron flasks, open Battersea crucibles, aluminum solder, and his aluminum bases Nos. 1 and 2, for upper and lower dentures respectively. As the result of a series of experiments, carried on for over twenty years, the aluminum of commerce is by his process freed from iron, silica, and other impurities, and rendered almost chemically pure, and then so alloyed with a noble metal as to overcome all shrinkage, furnishing a non-oxidizable base, having a polish like nickle-plate, and which combines in itself the greatest possible lightness, stiffness, strength, and durability. This metal is also perfectly compatible to the tissues of the oral cavity, and is entirely free from galvanic action if coming in contact with fillings of other metals.

Plates made from the aluminum heretofore furnished for dental purposes, disintegrate through the action of the fluids of the oral cavity; chemically pure aluminum also contracts to such an extreme degree that its use for dental purposes is impracticable. To overcome these difficulties Dr. Carroll has succeeded in alloying it with a 3 per centage of other metals, giving a permanent compound of 97 per cent. of chemically pure aluminum. For his cast-aluminum dental work, the impression is taken, preferably with plaster, as for any other method of plate work, the cast being made with sand and plaster; the bite is taken and the teeth mounted as usual, except that the moulding and carving must be very exact, as there should be no retouching after the metal is cast, beyond trimming off the surplus. The work is flasked the same as for vulcanite; either gum-section or plain teeth can be used, and either pink rubber or celluloid attachments, or casting the metal directly to the teeth if there is no exposure of the gums. The only point requiring special attention is that of spacing the sections slightly (as with a slip of paper) to prevent breakage from the slight degree of contraction which has not, as yet, in all cases, been entirely overcome. In using gum-sections the metal is not cast over the margin of the gum, but a line of No. 2 base is subsequently run over the edges with a soldering iron. This is rapidly and easily done and prevents liability of fracture. For celluloid attachments the piece is cast with an under cut flange and the celluloid pressed at a second operation. Dr. Carroll has discovered the means of soldering and welding aluminum, which has hitherto been considered an impossibility.

At the 8 to 10.30 Clinics, Thursday, Dr. B. Holly Smith (Baltimore) crowned, at a single operation, four badly decayed, but not broken down, superior incisors which had living pulps when he began the operation. The crowns were cut off, and while the pulps were numbed by the shock, the pulp tissue was at once removed with broaches. The patient apparently felt no pain from the removal of the pulps. The aqueous solution of hydro-napthol was used as an antiseptic wash, the roots filled, and the crowns immediately mounted.

Dr. T. S. Waters (Baltimore) made a compound contour filling in a superior central incisor (without the usual protection from thermal changes by oxyphosphate,) with No. 30 cohesive gold, and using the electric mallet, a description of the use of the latter being made a special feature of the operation. Gold fillings in adjacent teeth were protected from being scratched with a cap or matrix fitted over the crown, made in the same manner as Dr. Herbst made his matrices.

Dr. C. S. Case (Jackson, Mich.) filled an anterior proximal cavity in a lower molar using tin and gold foil and Robinson's felt-foil and gold mixed, the surface of the filling finished with a hard No. 60 rolled platinum and gold articulating face. He folds the felt-foil over gold and cuts into squares. He uses a heavy hand mallet and bridge-shaped pluggers and makes a large filling two-thirds tin and gold combined.

Dr. E. C. Moore (Detroit, Mich.) filled an anterior proximal cavity in a first bicuspid, using William's soft-foil cylinders, with the electric mallet.

Dr. C. A. Timme (Hoboken, N. J.) made a compound posterior and grinding surface filling in the first left inferior molar, using Wolrab's gold by the Herbst method.

Dr. S. B. Price (New York) filled an anterior proximal cavity in a superior second bicuspid using soft gold with smooth point pluggers.

Dr. E. S. Niles (Boston, Mass.) filled proximal cavities between a superior central and lateral incisor, using Wolrab's gold.

Dr. L. L. Davis (Eaton Rapids, Mich.) filled a superior right bicuspid, contouring and using the electro-magnetic mallet.

Dr. Wm. Crenshaw (Atlanta, Ga.) completed his series of eight or ten consecutive proximal fillings in the right upper jaw, beginning with second bicuspid, the teeth having been much cut away with files, etc., making large contour operations.

Dr. R. B. Adair continued his treatment of several cases of Pyorrhea Alveolaris.

FRIDAY, SEPT. 9.

Dr. T. S. Waters (Baltimore) exhibited in the mouth a practical case of removable bridgework, the piece being held in place by little springs set in the crowns and fitting the prepared roots of the natural teeth.

Dr. Wm. Barker (Providence, R. I.) contoured the mesial surface and restored the distal cusp of a first bicuspid, using cohesive and non-cohesive gold with hand pressure and the mallet.

Dr. R. H. Woodhouse (London, England) contoured a right superior first bicuspid.

Dr. Morrison (St. Louis) was preparing to fill a pulp canal by his well-known method of using gold wire when he encountered a live pulp in the central incisor which was not fully exposed ; he therefore capped the pulp with oxyphosphate, and put in a temporary filling.

Dr. W. H. Richards (Knoxville, Tenn.) demonstrated his method of treating central abscess.

Dr. G. D. Curtis (Syracuse, N. Y.) implanted a superior bicuspid for Dr. McLeod, Edinburg, Scotland.

Dr. G. A. Sprenkel (Culpepper, Va.) filled an proximal cavity in the superior left central, using soft foil and contouring.

Dr. E. L. Swartout (Utica, N. Y.) filled the labial surface of a right superior cuspid, lining walls of the cavity with Wolrab's gold and packing with Watt's Crystal gold.

Dr. J. R. Knapp (New Orleans, La.) placed in the mouth a porcelain faced superior bicuspid crown. The tooth had been filled with amalgam and was badly discolored ; the roots had never been treated or cleaned ; had been abscessed for years, accompanied by eruption on the face at the base of the nose.

Dr. G. W. Whitefield (Evanston, Ill.) demonstrated his method of bleaching by passing an electric current through a discolored tooth packed with chloride of sodium, using a double pointed broach insulated by an ivory ring, bleaching the dentine perfectly in 45 minutes.

Dr. R. B. Adair continued his Pyorrhea Alveolaris treatment, having been signally successful in the case of Chas. F. Gillis, whose left superior lateral incisor presented a typical case, with pockets reaching to the apex, and so loose that it could have been easily removed with the fingers. It had to be ligated before the deposits could be removed at the first sitting. The improvement was very marked on this, the third day of treatment.

Several operations were finished on Saturday morning, the day after adjournment, interest in the clinics being unabated.

