# Correction

**Published:** 1888-03

**Authors:** 


### Correction.

In the February number of the DENTAL REGISTER, page 84, second paragraph from the bottom, in the report of the Clinic of the Dental Section, 9th International Medical Congress, is the following sentence: "These arrangements were all planned and carried out by Dr. R. Finlay Hunt, Washington, D. C." This statement is not correct. It does injustice to other parties who are equally interested and engaged with Dr. Hunt in securing the arrangements for the work of the Section. Dr. Hunt was the Committee to secure buildings for the use of the Section. In this he succeeded admirably, and to the delight of everyone interested, and to this his efforts were chiefly confined. For the admirable arrangements for the work of the Section, credit is due chiefly to the Chairman of the Committee on Prosthetic Dentistry, Dr. H. B. Noble, together with the other members of his committee, namely; Drs. R. B. Donaldson, M. F. Finlay, and others.

Upon this committee devolved mainly the preparation of the rooms for both the Prosthetic and Operative departments. They received valuable aid, however, from Mr. Selby, of the S. S. White Co., and others, but upon the resident members of the committee devolved a large share of the work, for which the Section was greatly indebted.

